# Single-cell transcriptome atlas unveils transcriptional regulation networks of banana root tips in response to *Fusarium oxysporum* infection

**DOI:** 10.1093/hr/uhaf220

**Published:** 2025-08-22

**Authors:** Kaisen Huo, Meiying Li, Dongxu Lan, Xiaoxue Ye, Zhengnan Xie, Yan Yan, Wei Wang, Jianxiang Ma, Chaochao Li, Weiwei Tie, Wei Hu, Jianghui Xie, Zehong Ding

**Affiliations:** State Key Laboratory of Tropical Crop Breeding, Institute of Tropical Bioscience and Biotechnology, Coconut Research Institute, Sanya Research Institute, Chinese Academy of Tropical Agricultural Sciences, 4 Xueyuan Road, Haikou, China; Hainan Key Laboratory for Protection and Utilization of Tropical Bioresources, Hainan Institute for Tropical Agricultural Resources, Chinese Academy of Tropical Agricultural Sciences, 4 Xueyuan Road, Haikou, China; State Key Laboratory of Tropical Crop Breeding, Institute of Tropical Bioscience and Biotechnology, Coconut Research Institute, Sanya Research Institute, Chinese Academy of Tropical Agricultural Sciences, 4 Xueyuan Road, Haikou, China; Hainan Key Laboratory for Protection and Utilization of Tropical Bioresources, Hainan Institute for Tropical Agricultural Resources, Chinese Academy of Tropical Agricultural Sciences, 4 Xueyuan Road, Haikou, China; State Key Laboratory of Tropical Crop Breeding, Institute of Tropical Bioscience and Biotechnology, Coconut Research Institute, Sanya Research Institute, Chinese Academy of Tropical Agricultural Sciences, 4 Xueyuan Road, Haikou, China; Hainan Key Laboratory for Protection and Utilization of Tropical Bioresources, Hainan Institute for Tropical Agricultural Resources, Chinese Academy of Tropical Agricultural Sciences, 4 Xueyuan Road, Haikou, China; State Key Laboratory of Tropical Crop Breeding, Institute of Tropical Bioscience and Biotechnology, Coconut Research Institute, Sanya Research Institute, Chinese Academy of Tropical Agricultural Sciences, 4 Xueyuan Road, Haikou, China; Hainan Key Laboratory for Protection and Utilization of Tropical Bioresources, Hainan Institute for Tropical Agricultural Resources, Chinese Academy of Tropical Agricultural Sciences, 4 Xueyuan Road, Haikou, China; State Key Laboratory of Tropical Crop Breeding, Institute of Tropical Bioscience and Biotechnology, Coconut Research Institute, Sanya Research Institute, Chinese Academy of Tropical Agricultural Sciences, 4 Xueyuan Road, Haikou, China; Hainan Key Laboratory for Protection and Utilization of Tropical Bioresources, Hainan Institute for Tropical Agricultural Resources, Chinese Academy of Tropical Agricultural Sciences, 4 Xueyuan Road, Haikou, China; State Key Laboratory of Tropical Crop Breeding, Institute of Tropical Bioscience and Biotechnology, Coconut Research Institute, Sanya Research Institute, Chinese Academy of Tropical Agricultural Sciences, 4 Xueyuan Road, Haikou, China; Hainan Key Laboratory for Protection and Utilization of Tropical Bioresources, Hainan Institute for Tropical Agricultural Resources, Chinese Academy of Tropical Agricultural Sciences, 4 Xueyuan Road, Haikou, China; State Key Laboratory of Tropical Crop Breeding, Institute of Tropical Bioscience and Biotechnology, Coconut Research Institute, Sanya Research Institute, Chinese Academy of Tropical Agricultural Sciences, 4 Xueyuan Road, Haikou, China; Hainan Key Laboratory for Protection and Utilization of Tropical Bioresources, Hainan Institute for Tropical Agricultural Resources, Chinese Academy of Tropical Agricultural Sciences, 4 Xueyuan Road, Haikou, China; State Key Laboratory of Tropical Crop Breeding, Institute of Tropical Bioscience and Biotechnology, Coconut Research Institute, Sanya Research Institute, Chinese Academy of Tropical Agricultural Sciences, 4 Xueyuan Road, Haikou, China; Hainan Key Laboratory for Protection and Utilization of Tropical Bioresources, Hainan Institute for Tropical Agricultural Resources, Chinese Academy of Tropical Agricultural Sciences, 4 Xueyuan Road, Haikou, China; State Key Laboratory of Tropical Crop Breeding, Institute of Tropical Bioscience and Biotechnology, Coconut Research Institute, Sanya Research Institute, Chinese Academy of Tropical Agricultural Sciences, 4 Xueyuan Road, Haikou, China; Hainan Key Laboratory for Protection and Utilization of Tropical Bioresources, Hainan Institute for Tropical Agricultural Resources, Chinese Academy of Tropical Agricultural Sciences, 4 Xueyuan Road, Haikou, China; College of Agriculture, Guizhou University, Guiyang, China; State Key Laboratory of Tropical Crop Breeding, Institute of Tropical Bioscience and Biotechnology, Coconut Research Institute, Sanya Research Institute, Chinese Academy of Tropical Agricultural Sciences, 4 Xueyuan Road, Haikou, China; Hainan Key Laboratory for Protection and Utilization of Tropical Bioresources, Hainan Institute for Tropical Agricultural Resources, Chinese Academy of Tropical Agricultural Sciences, 4 Xueyuan Road, Haikou, China; State Key Laboratory of Tropical Crop Breeding, Institute of Tropical Bioscience and Biotechnology, Coconut Research Institute, Sanya Research Institute, Chinese Academy of Tropical Agricultural Sciences, 4 Xueyuan Road, Haikou, China; Hainan Key Laboratory for Protection and Utilization of Tropical Bioresources, Hainan Institute for Tropical Agricultural Resources, Chinese Academy of Tropical Agricultural Sciences, 4 Xueyuan Road, Haikou, China; Hainan Key Laboratory for Biosafety Monitoring and Molecular Breeding in Off-Season Reproduction Regions, Key Laboratory of Biology and Genetic Resources of Tropical Crops, Institute of Tropical Bioscience and Biotechnology, Chinese Academy of Tropical Agricultural Sciences, 4 Xueyuan Road, Haikou, China; State Key Laboratory of Tropical Crop Breeding, Institute of Tropical Bioscience and Biotechnology, Coconut Research Institute, Sanya Research Institute, Chinese Academy of Tropical Agricultural Sciences, 4 Xueyuan Road, Haikou, China; Hainan Key Laboratory for Protection and Utilization of Tropical Bioresources, Hainan Institute for Tropical Agricultural Resources, Chinese Academy of Tropical Agricultural Sciences, 4 Xueyuan Road, Haikou, China; Hainan Key Laboratory for Biosafety Monitoring and Molecular Breeding in Off-Season Reproduction Regions, Key Laboratory of Biology and Genetic Resources of Tropical Crops, Institute of Tropical Bioscience and Biotechnology, Chinese Academy of Tropical Agricultural Sciences, 4 Xueyuan Road, Haikou, China; State Key Laboratory of Tropical Crop Breeding, Institute of Tropical Bioscience and Biotechnology, Coconut Research Institute, Sanya Research Institute, Chinese Academy of Tropical Agricultural Sciences, 4 Xueyuan Road, Haikou, China; Hainan Key Laboratory for Protection and Utilization of Tropical Bioresources, Hainan Institute for Tropical Agricultural Resources, Chinese Academy of Tropical Agricultural Sciences, 4 Xueyuan Road, Haikou, China; Hainan Key Laboratory for Biosafety Monitoring and Molecular Breeding in Off-Season Reproduction Regions, Key Laboratory of Biology and Genetic Resources of Tropical Crops, Institute of Tropical Bioscience and Biotechnology, Chinese Academy of Tropical Agricultural Sciences, 4 Xueyuan Road, Haikou, China

## Abstract

Fusarium wilt caused by *Fusarium oxysporum* (*Foc*) is one of the most destructive diseases in global banana production. The response of root system to *Foc* infection through gene expression in multiple cell types is crucial for understanding the disease resistance mechanism in banana. Here, we reported a single-cell transcriptional landscape of banana root tips in response to *Fusarium oxysporum* f. sp. *cubense* tropical race 4 (*Foc* TR4) infection. We characterized 10 major cell types from 19 cell clusters. We explored differentiation trajectories of meristematic cells, root cap cells, and pericycle cells through pseudotime analysis, and identified pericycle cell as the dominant root cell type under *Foc* TR4 infection. Moreover, we identified 11 co-expression regulatory networks, of which eight were significantly associated with *Foc* TR4 response. Specifically, *MaKAN4* was co-expressed with two Zn ^2+^-dependent genes (*MaACA7* and *MaADH3*) in M5 module, which was associated with pericycle cell type and responded to *Foc* TR4 infection. Further analysis demonstrated that MaKAN4 protein could interact with the promoters of *MaACA7* and *MaADH3* to promote their expression levels, highlighting a crucial role of *MaKAN4* in banana disease resistance by regulating the Zn ^2+^-dependent *MaACA7*/*MaADH3* module. These findings provide a comprehensive view of cell fate determination in banana root tips and highlight novel insights into the regulatory mechanisms of major cell types in response to *Foc* TR4 infection, laying a useful foundation for developing disease-resistant banana cultivars.

## Introduction

Banana (*Musa* spp.) is a staple food for over 400 million people, with over 40% of global production and nearly all export trade relying on Cavendish bananas [1]. Fusarium wilt is one of the most devastating banana diseases worldwide, causing a serious threat to the global banana industry [2]. *Foc* TR4, one of the most widespread hosts among different *Foc* biotypes, can infect the main commercial cultivar ‘Cavendish’ banana, and is therefore the most aggressive causal agents of bananas in recent years [3]. *Foc* TR4 can be spread through various channels including sucking buds, bacterial soils, rainwaters, farming tools, and diseases remain in the field [4, 5]. *Foc* TR4 can directly infect banana roots and colonize the rhizome vasculature, then continue to spread upward throughout the plant [6]. Previous studies indicate that two transgenic Cavendish lines with the *RGA2* and *Ced9* genes exhibit disease resistance, as evidenced by a 3-year field trial [1].

Roots help vascular plants adapt to environmental challenges, especially as the first tissue to be directly infected by soil-borne pathogens [7]. In recent years, the defense mechanism against banana Fusarium wilt has been studied by various omics approaches [8–10]. Comparative transcriptome analysis between resistant and susceptible bananas revealed that differentially expressed genes (DEGs) were mainly enriched in plant–pathogen interaction and plant hormone signal transduction pathways. Defense genes related to NB-LRR proteins, PR proteins, ethylene-responsive transcription factors, and lignin biosynthesis were actively upregulated in response to Fusarium wilt treatment [3, 9, 10]. Notably, the resistant banana cultivar ‘Goldfinger’ (AAAB) had fewer aerenchyma compared to the susceptible cultivar ‘Cavendish’ (AAA), which limited oxygen availability for the pathogen and thereby restricted its respiration, survival, and vertical invasion in vascular [11]. These results suggested that specific root tissues played a crucial role in banana’s defense response to Fusarium wilt.

Cellular heterogeneity is important for understanding the complex gene regulatory network of roots under biotic stresses [12]. However, conventional omics may obscure the characteristics of different cell types. For this reason, single-cell RNA sequencing (scRNA-seq) technology has been applied to plant biology, completing high-resolution mapping of transcriptional landscapes in roots of *Arabidopsis thaliana*, rice, and maize [13–15]. ScRNA-seq has also been utilized to investigate expression signatures of major cell types responding to biotic stresses in rubber trees [16], strawberries [17], and maize [18], but there are currently no applications of scRNA-seq in banana.

Here, we performed scRNA-seq analysis of Cavendish root tips to detect cell-specific responses under *Foc* TR4 infection. These findings reveal a single-cell transcriptional atlas of banana root tips and provide novel insights into the regulatory mechanisms of major cell types in response to *Foc* TR4 infection, laying a useful foundation for developing disease-resistant banana cultivars.

## Results

### Cell type observation in banana root tips under *Fusarium oxysporum* infection

**Figure 1:**
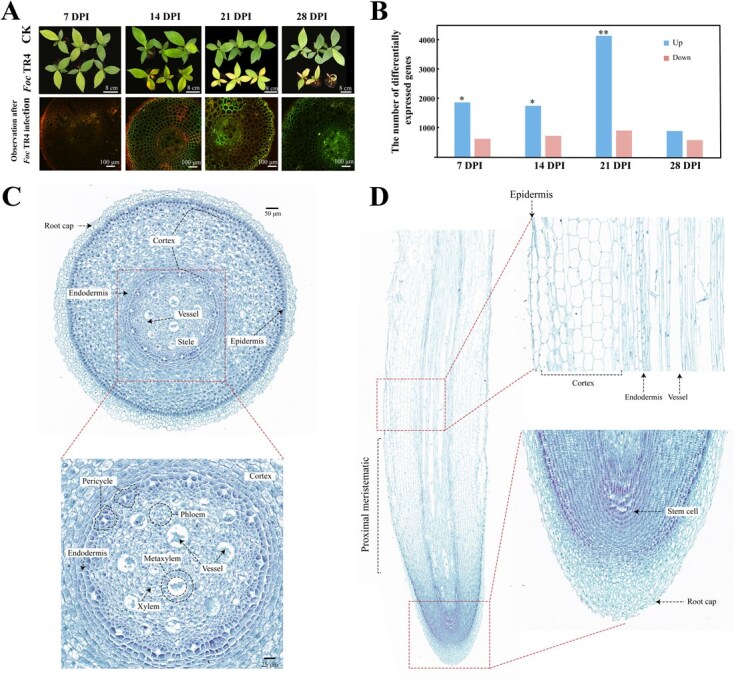
The infection of *Fusarium oxysporum* in banana seedling roots and microscopic observation of cell types. (A) Phenotypic observation and confocal laser microscopy of root tips after *Foc* TR4 infection. Green fluorescence indicates GFP-labeled *Foc* TR4. (B) Number of differentially expressed genes in banana root tips after *Foc* TR4 infection using bulk RNA-seq. * and ** indicate P < 0.05 and P < 0.01, respectively. (C) Observation of cell types in transverse sections of banana root tips. (D) Observation of cell types in longitudinal sections of banana root tips.

Overall, the *Foc* TR4-infected plants remained generally healthy at 7 days post inoculation (DPI, Fig. 1A). Compared with the control, the leaves of *Foc* TR4-treated plants were yellowing at 14–21 DPI and wilted at 28 DPI (Fig. 1A). Laser confocal microscopy showed that *Foc* TR4 had completely colonized in root tips at 21 DPI (Fig. 1A). Bulk RNA sequencing showed that the number of DEGs was peaked at 21 DPI in root tips under *Foc* TR4 infection (Fig. 1B). Together, these results indicate that 21 DPI is the optimal time point for further analysis of banana root tips in response to *Foc* TR4 infection.

Paraffin section of root tips at 21 DPI revealed that the cell types included root cap, cortex, endodermis, pericycle, vessel, phloem, xylem, metaxylem, proximal meristematic, and stem cells, providing a general foundation for the subsequent screening of cell-specific marker genes (Fig. 1C-D).

### Nucleus isolation and scRNA-seq

**Figure 2:**
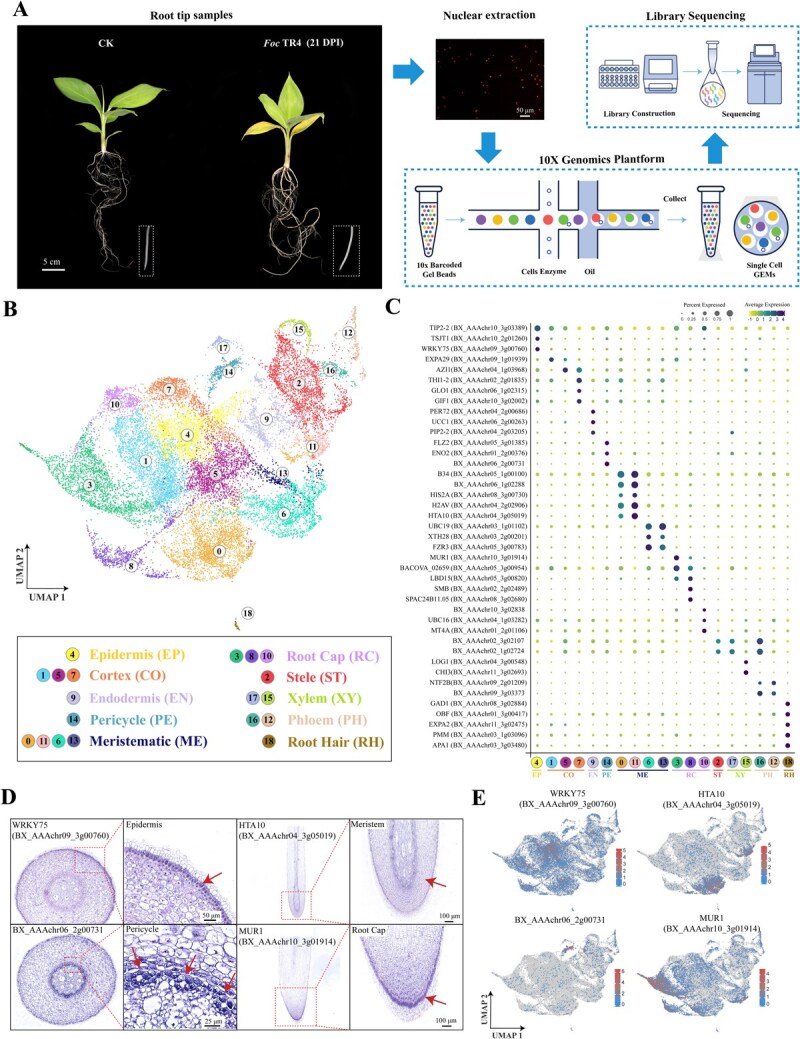
A single-cell transcriptome atlas of banana roots tips under *Foc* TR4 infection. (A) scRNA-seq of Cavendish banana root tips was performed using the 10X Genomics Chromium platform. (B) Visualization of 19 cell clusters and their spatial distribution in root tips using UMAP. (C) Expression patterns of marker genes for ten cell types. Dot size indicates the proportion of cells expressing the gene, and color represents the average gene expression level. (D) RNA *in situ* hybridization of cell-type marker genes for four cell types. (E) Visualization of specific expression of four marker genes by UMAP. Gene expression levels are shown from blue (low) to red (high).

We used optimized dissociation methods to isolate high-quality single-cell nuclear suspensions, from which total RNA was extracted for scRNA-seq on the 10x Genomics platform (Fig. 2A). Two groups of banana root tips were performed: CK (natural growth) and *Foc* TR4 (21 days post *Foc* TR4 inoculation). A total of 48 983 nuclei and ~ 3.9 million reads per transcriptome were obtained by efficient cell identification and unique molecular identifier (UMI) correction (Supplementary Table S1). After removing low-quality cells, the average median number of UMIs per cell was 7529 and the average median number of genes per cell was 6291 (Supplementary Fig. S1, Supplementary Table S2 and S3). After linear dimensionality reduction, the 48 983 nuclei were filtered into 44 306 cells and further divided to 19 cell clusters (Supplementary Fig. S2).

### Single-cell atlas of banana root tips

We annotated the cell clusters using homologs of *Arabidopsis* cluster-specific genes (Supplementary Table S4), because of the absence of previous reports on banana cell heterogeneity. PlantCellMarker (https://www.tobaccodb.org) was used to search for specific marker genes from published scRNA-seq data to help prediction of cell types. Using marker genes, we grouped 19 clusters into 10 cell types, including cortex, endodermis, epidermis, pericycle, meristem, root cap, stele, xylem, phloem, and root hair (Fig. 2B-C). We performed RNA *in situ* hybridization to identify major marker genes that could distinguish four cell types (including epidermis, pericycle, meristem, and root cap) of root tips (Fig. 2D). Consistently, four marker genes, including *BX_AAAchr09_3g00760 (WRKY75)*, *BX_AAAchr06_2g00731*, *BX_AAAchr04_3g05019 (HTA10)*, and *BX_AAAchr10_3g01914 (MUR1)*, showed cell-specific expression in epidermis, pericycle, meristem, and root cap by the uniform manifold approximation and projection (UMAP), respectively (Fig. 2E).

In meristematic cells, *B34* (*BX_AAAchr05_1g00100*) was identified as a marker gene in clusters 0 and 11 (Fig. 2C and Supplementary Table S4), and its homolog was identified as a marker for meristematic cells in *Arabidopsis* [19]. Another two histone-related genes, *HIS2A* (*BX_AAAchr08_3g00730*) and *H2AV* (*BX_AAAchr04_2g02906*), were also identified as marker genes in clusters 0 and 11 (Fig. 2C). Their homologs were found to serve as markers for early-stage meristem cells in *Arabidopsis* scRNA-seq data [20, 21]. Previous studies have indicated that these histone-related genes played a crucial role as epigenetic regulators in root meristems [22]. RNA *in situ* hybridization identified *HTA10* (*BX_AAAchr04_3g05019*) as a marker for meristem in clusters 0 and 11 (Fig. 2D and Supplementary Table S4). Meanwhile, *UBC19* (*BX_AAAchr03_1g01102*), *XTH28* (*BX_AAAchr03_2g00201*), and *FZR3* (*BX_AAAchr05_3g00783*) were specifically expressed in clusters 6 and 13 (Fig. 2C), and their homologs have been identified as marker genes of root meristematic cells in *Arabidopsis* [23–25].

In root cap cells, marker genes *MUR1* (*BX_AAAchr10_3g01914*), *BACOVA_02659* (*BX_AAAchr05_3g00954*), and *LBD15* (*BX_AAAchr05_3g00820*) were specifically expressed in clusters 3 and 8. Their homologs have been identified as specific markers of root cap cells in *Arabidopsis* [20, 24, 25]. RNA *in situ* hybridization indicated *MUR1* as the maker gene of a transitional cell type between root apical meristem and root cap, exhibiting a cap-like distribution. Moreover, the process of cell elongation was observed during the transition from root apical meristem to root cap in this work (Fig. 2D), in accord with the roles of *MUR1* in cell elongation [26]. Therefore, we defined clusters 3 and 8 as the inner root cap (Fig. 2D). *UBC16* (*BX_AAAchr04_1g03282*) and *MT4A* (*BX_AAAchr01_2g01106*) were identified as marker genes in cluster 10 (Fig. 2C), in accord with that their homologs were identified as marker genes of root cap cells in *Arabidopsis* scRNA-seq data [27, 28]. Here, we designated cluster 10 as the outer root cap to distinguish it from the inner root cap.

In root hair cells, two marker genes, *GAD1* (*BX_AAAchr08_3g02884*) and *OBF* (*BX_AAAchr01_3g00417*), were specifically expressed in clusters 18. Their homologs were recognized as marker genes of root hair in *Arabidopsis* [13, 25]. The homologs of another three marker genes, including *EXPA2* (*BX_AAAchr11_3g02475*), *APA1* (*BX_AAAchr03_3g03480*) and *PMM* (*BX_AAAchr03_1g03096*), have also been identified as marker genes in root hairs of *Arabidopsis* [25, 29, 30]. They were also identified as markers for *Arabidopsis* trichoblast cells, a specific cell type involved in the differentiation from epidermal cells to root hair cells [25, 31].

In epidermal cells, marker genes *TIP2–2* (*BX_AAAchr10_3g03389*) and *TSJT1* (*BX_AAAchr10_2g01260*) were specifically expressed in cluster 4. Additionally, the transcription factor *WRKY75* (*BX_AAAchr09_3g00760*) was specifically expressed in cluster 4, and its homolog has been recognized as a marker gene for epidermal cells in *Arabidopsis* [14, 25, 32].

In cortex cells, *EXPA29* (*BX_AAAchr09_1g01939*) was specifically expressed in clusters 1 and 5, and its homolog was recognized as a specific marker gene for root cortex cells in *Arabidopsis* [33]. The lipid transfer protein-encoding gene *AZI1* (*BX_AAAchr04_1g03968*), whose homolog mediated signal mobilization for systemic defense priming in root cortex cells in *Arabidopsis* [34], was highly expressed in clusters 5 and 7. Other marker genes, including *THI1–2* (*BX_AAAchr02_2g01835*), *GLO1* (*BX_AAAchr06_1g02315*), and *GIF1* (*BX_AAAchr10_3g02002*), were exclusively expressed in cluster 7, and their homologs were identified as specific marker genes of cortex cells in *Arabidopsis* roots [35–37].

In endodermal cells, two marker genes, *PER72* (*BX_AAAchr04_2g00686*) and *UCC1* (*BX_AAAchr06_2g00263*), were specifically expressed in cluster 9, and their homologs have been implicated in lignin polymerization at root endodermis of *Arabidopsis* [38, 39]. The homolog of another marker gene identified here, *PIP2–2* (*BX_AAAchr04_2g03205*), has been recognized as a marker for endodermal cells in *Arabidopsis* [29, 35]. Moreover, its homolog from *Glycine max* was specifically expressed in endodermal cells of root nodules [40].

Stele cells could not be clearly distinguished from xylem cells and phloem cells as they were closely related. This was evidenced by the specific expression of *BX_AAAchr02_1g02724* and *BX_AAAchr02_3g02107* in clusters 2, 17, and 16 (Fig. 2C). Moreover, the homolog of *BX_AAAchr02_1g02724* was identified as a marker gene of stele in *Arabidopsis* root [41]. However, phloem cells and xylem cells could be clearly distinguished. The marker genes *NTF2B* (*BX_AAAchr09_2g01209*) and *BX_AAAchr09_3g03373* were specifically expressed in clusters 16 and 12, and their homologs have been recognized as marker genes for phloem in *Arabidopsis* root [21, 42]. *CHI3* (*BX_AAAchr11_3g02693*) was specifically expressed in cluster 15, and its homolog was identified as a representative marker gene of xylem cells in *Arabidopsis* [21]*.*

Overall, UMAP projection analysis identified ten cell types based on 101 specific marker genes (Supplementary Table S4): root meristematic cells (clusters 0, 6, 11, and 13), root cap cells (clusters 3, 8, and 10), root hair cells (cluster 18), epidermal cells (cluster 4), cortex cells (clusters 1, 5, and 7), stele cells (cluster 2), phloem cells (clusters 12 and 16), endodermis cells (cluster 9), pericycle cells (cluster 14), and xylem cells (cluster 15 and 17).

### Differentiation trajectories of meristematic cells

The common origin of different cell types facilitates a highly integrated differentiation process, enhancing adaptability to diverse soil environments [43]. In the following, pseudotime analysis was performed to explore the differentiation order and fate of meristematic cells and root cap cells, respectively.

**Figure 3:**
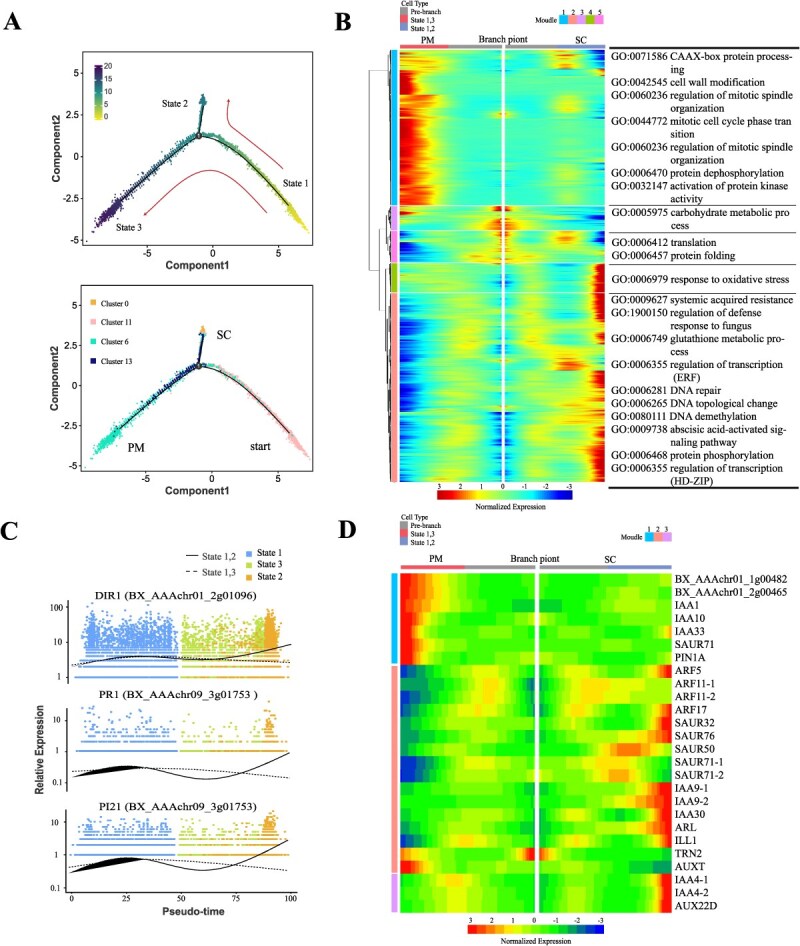
Pseudotime trajectory of meristem upon *Foc* TR4 infection. (A) Pseudotime developmental trajectory of meristematic cells. In the pseudotime trajectory, lighter colors indicate earlier developmental stages. (B) The heatmap shows the differentially expressed genes in stem cells (SC) and proximal meristem (PM) branches during the differentiation trajectory. Gene functions are revealed by GO enrichment. (C) Pseudotime expression pattern of disease resistance-related genes. (D) Heatmap of differentially expressed genes related to auxin.

Meristematic cells contained stem cells and proximal meristem cells that were clustered at opposite ends of the pseudotime backbone (Fig. 3A). We observed that clusters 0 and 11 mainly occupied state 1 and 2, while clusters 6 and 13 distributed in state 3 (Fig. 3A). By analyzing branch-dependent genes with a heatmap, we identified five distinct expression modules. The fate-determining genes of modules 1, 2, and 4 were distinctly located at the ends of the differentiation trajectory, while modules 3 and 5 were in the intermediate stages (Fig. 3B). Proximal meristem-related genes were predominantly found in module 1, which was significantly enriched in GO terms such as GO:0032147 (activation of protein kinase activity), GO:0044772 (mitotic cell cycle phase transition), and GO:0060236 (regulation of mitotic spindle organization) (Supplementary Table S5). These GO biological processes were known to be crucial in proximal meristem cells for promoting root development [44–46]. Stem cell-related genes were mainly found in module 2, which showed significant enrichment in GO terms like GO:0006281 (DNA repair), GO:0006265 (DNA topological change), and GO:0080111 (DNA demethylation). These GO terms were associated with chromatin regulation and DNA repair, actively influencing stem cell fate [47–49]. Notably, the delay infection gene *PI21*, the systemic acquired resistance-related gene *DIR1*, and the pathogenesis-related gene *PR1* were prominent in state 2 at the later stage, exhibiting a similar expression pattern (Fig. 3C and Supplementary Fig. S3). Of which, *PR1* gene served as a marker for the salicylic acid (SA) pathway, and its promoter region contained several typical auxin response elements (including TGTC and TGTCT) [50, 51]. Then, we generated a heatmap of auxin-related DEGs along the pseudotime trajectory. A dozen of IAA and ARF genes, which were key regulators of *PR1* [52], were identified in module 2 (Fig. 3D). We hypothesized that ARF transcription factors directly regulated *PR1* expression, with a synergistic response of SA and auxin to *Foc* TR4 infection (Supplementary Fig. S4).

### Differentiation trajectories of root cap cells

**Figure 4:**
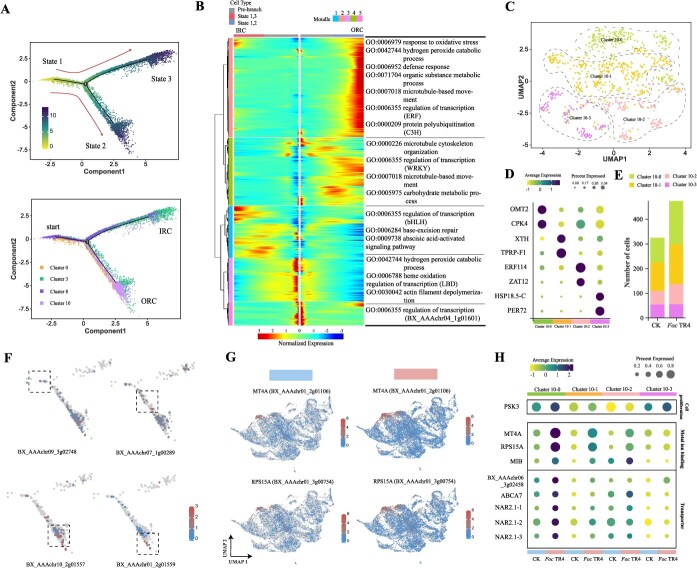
Pseudotime trajectory of root cap upon *Foc* TR4 infection. (A) Pseudotime developmental trajectory of root cap cells. In the pseudotime trajectory, lighter colors indicate earlier developmental stages. (B) The heatmap shows the differentially expressed genes in outer root cap (ORC) and inner root cap (IRC) during the differentiation trajectory. Gene functions are revealed by GO enrichment. (C) Re-clustering analysis of the ORC (cluster 10). (D) Specific marker genes identified from the re-clustering analysis of the ORC. (E) Cell frequency of four subclusters between the control and *Foc* TR4 treatment. (F) Expression atlas of specific marker genes in the pseudotime developmental trajectory after re-clustering of ORC. (G) Examples of genes specifically upregulated in ORC, visualized using UMAP in the control and *Foc* TR4 treatment samples. (H) Specific expression patterns of key genes in ORC responding to *Foc* TR4 infection.

We applied pseudo-time analysis on the cells from clusters 0, 3, 8, and 10 to investigate the differentiation of root cap cells. The cells from cluster 0 were differentiated into two trajectories, with clusters 3 and 8 located in the inner root cap while cluster 10 in the outer root cap (Fig. 4A). Branch-dependent genes were analyzed with a heatmap, and five expression modules were identified. The fate-determining genes of module 2 were located at the ends of the differentiation trajectory, while the remaining modules were in the intermediate stages (Fig. 4B). In module 2, outer root cap-related genes were involved in immune response, root development, and environmental sensing, and they were enriched in GO terms such as GO:0042744 (hydrogen peroxide catabolic process), GO:0006952 (defense response), and GO:0006979 (response to oxidative stress) (Supplementary Table S6). After *Foc* TR4 inoculation, the number of cells in cluster 10 exhibited the largest change, increasing by 50.43%. Therefore, cluster 10 was further subdivided into four subgroups based on the specific expression of marker genes (Fig. 4C-D). Cell frequency analysis found that after *Foc* TR4 infection, cluster 10–0 exhibited the greatest increase in cell number, followed by cluster 10–1 and cluster 10–2, while cluster 10–3 remained nearly unchanged (Fig. 4E). Both the slingshot analysis and the pseudo-time trajectory indicated that cluster 10–0 represented the initial outer root cap cells (Fig. 4F and Supplementary Fig. S5). These results suggested that the proliferation of outer root cap cells played a key role in response to *Foc* TR4 infection. *MT4A* and *RPS15A* were differentially upregulated in root cap cells after *Foc* TR4 infection compared with the control (Fig. 4G). They were associated with metal ion binding (GO:0046872 metal ion binding) and identified as upregulated marker genes in cluster 10–0 (Fig. 4H). Other upregulated marker genes in cluster 10–0, including *MIB*, *BX_AAAchr06_3g02458*, *ABCA7*, and *NAR2.1*, also played key roles related to metal ion binding and transporters (Supplementary Table S7). The marker gene *PSK3*, which was involved in cell proliferation, was significantly upregulated after *Foc* TR4 inoculation (Fig. 4H). Together, these findings suggested that the banana outer root cap, as the outermost layer of cells in directly contact with and sensing soils, responded to *Foc* TR4 infection by producing more initial outer root cap cells and actively regulating metal ions.

### The pericycle is the dominant cell type in response to *Foc* TR4 infection

**Figure 5:**
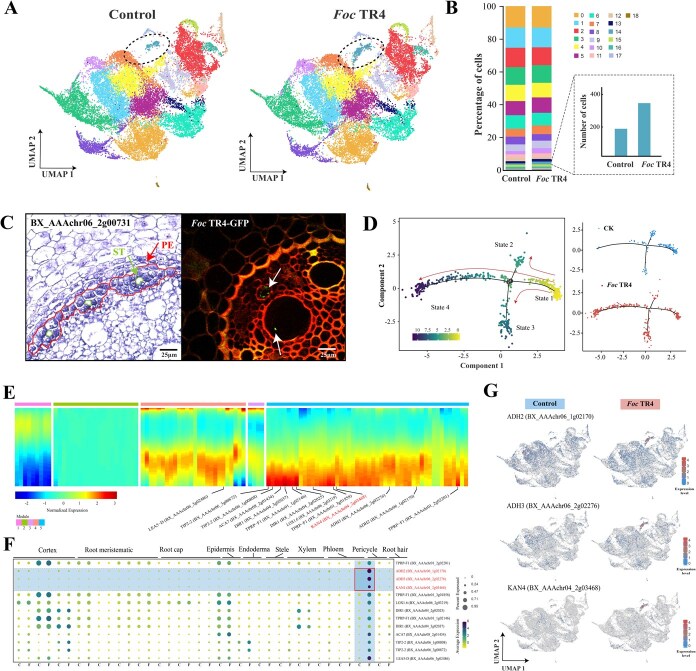
Pseudotime trajectory of pericycle upon *Foc* TR4 infection. (A) UMAP visualization of distinct cell types in root tips between the control and *Foc* TR4 treatment. (B) Cell frequency analysis between the control and *Foc* TR4 treatment. (C) Diagram showing the position of pericycle cells (PE) and *Foc* TR4-colonized stele (ST). (D) Pseudotime analysis of pericycle cells under *Foc* TR4 infection. In the pseudotime trajectory, lighter colors indicate earlier developmental stages. (E) Heatmap showing the expression of pseudotime DEGs from state 1 to state 4. A few key representatives were annotated in the heatmap. (F) Expression patterns of the key representatives mentioned in (E). (G) UMAP visualization of pericycle-specific expression genes between the control and *Foc* TR4 treatment.

In the UMAP plot, cluster 14 emerged as a cluster with dramatically increased cell numbers after *Foc* TR4 infection (Fig. 5A). Although the number of pericycle cells accounted for only about 2% of the root tip cells, it had the highest number of *Foc* TR4-responsive differential genes. Cell frequency analysis revealed that the number of pericycle cells increased by 88.38% in response to *Foc* TR4 infection (Fig. 5B). RNA *in situ* hybridization of the marker gene (*BX_AAAchr06_2g00731*) of cluster 14 and laser confocal both indicated that pericycle cells were the most adjacent and dominant cell type in response to *Foc* TR4 colonization (Fig. 5C). Pseudotime analysis of cluster 14 showed primary differentiation from state 1 into three main branches: states 2, 3, and 4. Compared to the control, states 3 and 4 exhibited significantly more differentiated cells in response to *Foc* TR4 infection (Fig. 5D). By analyzing branch-dependent genes using a heatmap, we identified five distinct expression modules from state 1 to 4 (Fig. 5E). The fate-determining genes of module 5 were distinctly located at the ends of the differentiation trajectory, while module 4 was in the intermediate stage. The overlap of branch-dependent genes and the top 100 DEGs was annotated in the heatmap as representatives in the pericycle (Fig. 5E, Supplementary Table S8 and S9). Of which, three key genes (including *KAN4*, *ADH2*, and *ADH3*) showed pericycle-specific expression by the UMAP projection (Fig. 5F-G).

### Cell type-specific responses to *Foc* TR4 infection

Differential expression analysis was performed between *Foc* TR4 treatment and the control conditions to identify *Foc* TR4-responsive genes. A total of 3715 DEGs were identified, including 1885 upregulated and 1830 downregulated genes (Supplementary Table S9). The pericycle, outer root cap, and epidermis were the cell types with the highest number of DEGs (Supplementary Fig. S6). The expression of most marker genes was significantly changed in their respective cell types upon *Foc* TR4 infection. For example, *FLZ2* and *ENO2* were significantly up-regulated in pericycle cells after *Foc* TR4 infection, while *WRKY75* was downregulated in epidermal cells (Supplementary Fig. S7A). Notably, several defense-associated genes exhibited significant differential expression in response to *Foc* TR4 infection. Such as *PI21*, associated with the regulation of defense responses to fungus, was significantly upregulated in both epidermal and pericycle cells (Supplementary Fig. S7B). *CPK4* and *CPK2*, involved in the plant-pathogen interaction, were significantly upregulated in outer root cap and pericycle cells, respectively (Supplementary Fig. S7C). KEGG analysis showed that *Foc* TR4-induced DEGs were significantly enriched in phenylalanine, linoleic acid metabolism, and ribosome circadian rhythm (Supplementary Fig. S8). Collectively, different cell types in root tips exhibited distinct gene-specific responses under *Foc* TR4 infection, highlighting a complexity of immune response as a coordinated multicellular regulatory mechanism.

### Co-expression regulatory modules in responsive to *Foc* TR4 infection

**Figure 6:**
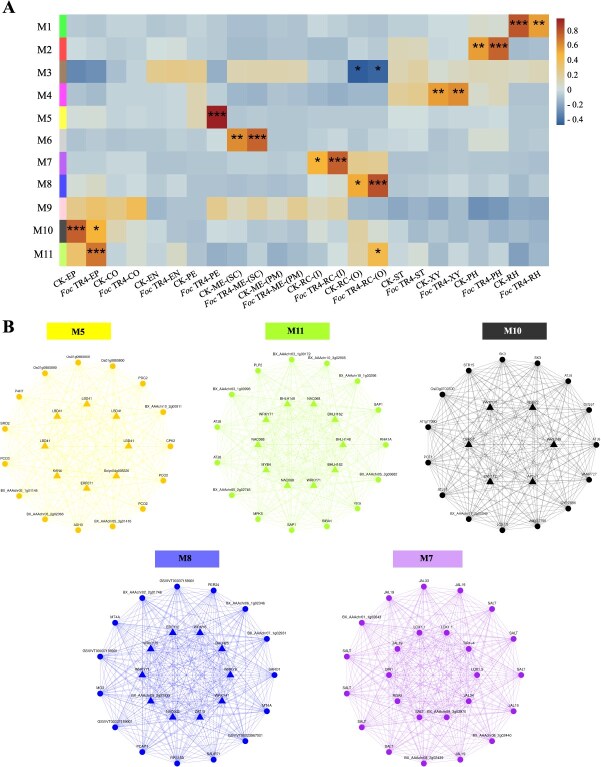
Co-expression modules of 10 major cell types in response to *Foc* TR4 infection. (A) 11 co-expression modules associated with cell types of root tips under *Foc* TR4 infection. The p-value is used to assess the significance of spatial autocorrelation. *, **, *** indicate P < 0.05, P < 0.01, and P < 0.001, respectively. (B) Five representative modules that are responsive to *Foc* TR4 infection. Each node represents a differentially expressed gene, and each triangle represents a differentially expressed transcription factor.

Gene co-expression network analysis was performed on the 3715 DEGs to identify co-expressed regulatory modules by setting soft-threshold = 15 (Supplementary Fig. S9). The co-expression network consisted of 11 modules (M1-M11), of which ten modules (M1-M8 and M10-M11) were significantly associated with different cell types and eight modules (M1, M2, M5, M6, M7, M8, M10, and M11) were significantly correlated with *Foc* TR4 infection and the control conditions, respectively (Fig. 6A and Supplementary Table S10). Among these *Foc* TR4-responsive modules, all were specific to a single cell type except for M11, which was expressed in both epidermis and outer root cap cells (Fig. 6A). For example, M5 module was significantly upregulated in pericycle cells after *Foc* TR4 infection. The genes from this module were enriched in multiple GO terms (GO:0016491, GO:0016702, and GO:0016705) related to oxidoreductase activity (Supplementary Fig. S10). Similarly, M8 and M7 were significantly upregulated in outer and inner root cap cells, respectively, in response to *Foc* TR4 infection. The M8 module was enriched in metal ion binding (GO:0046872) and cation binding (GO:0043169), while the M7 module was enriched in oxidative related processes (GO:0072593 and GO:0006979, Supplementary Fig. S10).

Core transcription factors and key genes were identified in eight *Foc* TR4-responsive modules by integrating connectivity and expression levels (Supplementary Table S11). The top five modules having most significant expression differences between the control and *Foc* TR4-treatment were displayed (Fig. 6B). Module M5 was specifically responded to *Foc* TR4 infection, with its gene network including hypoxia-related genes such as *ADH3*, *PCO2*, *PCO3*, and *KAN4* (Fig. 6B). Modules M7 and M8 were specifically responded in root cap. The gene network of M8 included metal ion-binding genes such as *MT4A* and *RPS15A*, consistent with our differential pseudotime analysis of the outer root cap (Fig. 4G-H). The gene network of M7 contained *JAL19*, *JAL33*, and *JAL34* (Fig. 6B), which encoded mannose-binding lectin proteins with antifungal activity and played a key role in response to *Foc* TR4 infection [53]. Collectively, we constructed regulatory networks of banana root tips in response to *Foc* TR4 infection, highlighting the synergistic response of multiple cell types and emphasizing a key role of pericycle cells in resisting *Foc* TR4 infection.

### MaKAN4 regulates Zn^2+^-dependent *MaACA7*/*MaADH3* module in pericycle cells responding to *Foc* TR4 infection

**Figure 7:**
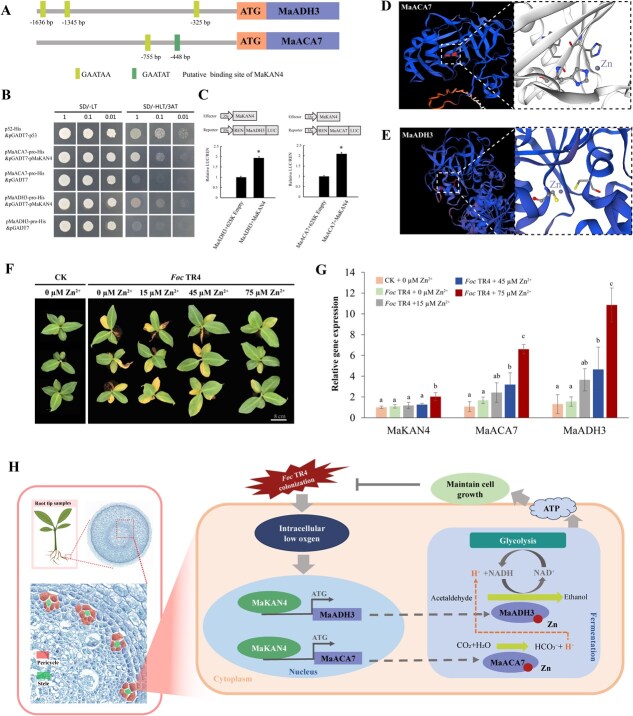
Molecular mechanism of MaKAN4 regulating Zn^2+^-dependent *MaACA7*/*MaADH3* module in pericycle cells under *Foc* TR4 infection. (A) Distribution of MaKAN4 binding sites in the promoters of *MaACA7* and *MaADH3*. (B) Yeast one-hybrid assay confirming the interaction between MaKAN4 and the promoters of *MaACA7* and *MaADH3*. (C) Dual-luciferase assay confirming the interaction between MaKAN4 and the promoters of *MaACA7* and *MaADH3*. (D) Binding site of Zn^2+^ with the imidazole ring of histidine in *MaACA7*. (E)Binding site of Zn^2+^ with the thiol group of cysteine in *MaADH3*. (F) Banana seedlings inoculated with *Foc* TR4 were treated with various concentrations of Zn^2+^ and the phenotypes were observed at 21 DPI. (G) Relative expression levels of *MaKAN4*, *MaACA7*, and *MaADH3* under various concentrations of Zn^2+^. Different lowercase letters indicate significant differences at P < 0.05 by one-way analysis of variance (ANOVA) with Tukey’s honestly significant difference test. (H) Schematic diagram of MaKAN4 regulating Zn^2+^-dependent *MaACA7*/*MaADH3* module in pericycle cells under *Foc* TR4 infection.

Both pseudotime and co-expression network analysis highlighted an important role of pericycle cells in response to *Foc* TR4 infection (Fig. 5E and Fig. 6B). *MaKAN4*, as a core transcription factor in the pericycle-specific module M5, was further investigated. Element analysis identified conserved regulatory elements (GAATAA and GAATAT) of MaKAN4 in the promoters of *MaACA7* and *MaADH3* (Fig. 7A). Yeast one-hybrid and dual-luciferase assays further confirmed that MaKAN4 interacted with the promoters of *MaACA7* and *MaADH3* (Fig. 7B-C). Interestingly, protein sequences of both MaACA7 and MaADH3 contained zinc binding site, suggesting that zinc ions might play a key role in pericycle cells in response to *Foc* TR4 infection (Fig. 7D-E). These findings supported a positive role of Zn^2+^ in defense against *Foc* TR4 infection via the *MaKAN4*-*MaACA7*/*MaADH3* module.

To investigate the roles of Zn^2+^ in defense against *Foc* TR4 infection, banana seedlings inoculated with *Foc* TR4 were treated with various concentrations of Zn^2+^ and the phenotypes were observed at 21 DPI (Fig. 7F). As expected, banana seedlings were gradually recovered with increasing Zn^2+^ concentrations. Moreover, the expression levels of *MaKAN4*, *MaACA7*, and *MaADH3* were significantly upregulated along with increased Zn^2+^ concentrations (Fig. 7G and Supplementary Table S12). These findings supported a positive role of Zn^2+^ in defense against *Foc* TR4 infection via the MaKAN4-MaACA7/MaADH3 module in perspective of phenotype and gene expression.

*ADH* genes were hypoxia-responsive during fungal colonization in plants [54–56]. ACA7, as an alpha carbonic anhydrase, was also hypoxia-responsive and provided H^+^ for the reduction of acetaldehyde [57, 58]. Another two hypoxia-inducible genes, *PCO2* and *PCO3* functioning as oxygen sensors [59], were upregulated in pericycle cells in this work (Supplementary Table S9). The upregulation of those hypoxia-responsive genes strongly suggested that pericycle cells were subjected to hypoxic stress under *Foc* TR4 infection. Collectively, these results revealed a hypoxia response mechanism of banana root tips responding to Fusarium infection, with MaKAN4 regulating *MaADH3* and *MaACA7* in the pericycle cells (Fig. 7H).

## Discussion

Banana Fusarium wilt, caused by the fungal pathogen *Foc*, is a devastating soil-borne disease that severely impacts global banana production [60]. Understanding the stress responses and developmental trajectories of different cell types using single-cell sequencing is crucial for deciphering the molecular mechanisms underlying banana Fusarium wilt resistance, as a few specific expression patterns are only observed in certain cell types [18]. To avoid interference with protoplast preparation and chloroplast RNA contamination, single nuclei are usually extracted to represent single cells for transcriptome sequencing [61]. This approach may result in the loss of cytoplasmic mRNAs and lowly expressed genes, but it improves the reliability and integrity of the transcriptomic data. Here, we reported the first comprehensive single-cell atlas of banana root tips, identifying 19 cell clusters and 10 major cell types using our defined cell marker genes (Supplementary Table S4), RNA *in situ* hybridization, and previously reported data [13, 62]. Different cell types exhibited specific expression responses to *Foc* TR4 infection, in which the pericycle had the highest number of DEGs. Notably, we identified a pericycle-specific marker gene, *BX_AAAchr06_2g00731*, in banana (Fig. 2D). Meanwhile, we identified that root meristem and root cap contained two subcellular types, which exhibited distinct cellular trajectories and different responsive patterns under *Foc* TR4 infection (Fig. 3 and Fig. 4).

A few resistance-related genes (*DIR1*, *PRI*, and *PI21*) were upregulated in stem cells as differentiation fate-associated genes (Fig. 3C). *TaDIR1–2*, the ortholog of *DIR1* in wheat, has been reported to play a negative regulatory role in resistance to *Puccinia striiformis* f. sp. *tritici* [63]. This suggests that *DIR1* may function as a potential regulator of defense responses, and thus its roles in banana responding to *Foc* TR4 infection deserve further investigation. IAAs inhibited ARF activity and provided feedback regulation in auxin signaling, while salicylic acid (SA) had antagonistic effects on auxin [52, 64]. Consistently, lots of ARF and IAA members were upregulated in stem cells in this work (Fig. 3D). Therefore, we proposed a potential co-regulatory model for SA and auxin, offering new insights into disease resistance in stem cells of banana root tips (Supplementary Fig. S4).

As the cell type directly interacting with Fusarium wilt in soils, metal ion-binding genes *MT4A* and *RPS15A*, along with multiple metal ion transport-related genes, were upregulated in root cap cells (Fig. 4H). In *Arabidopsis*, expression of the *MT4A* gene in vegetative tissues at various developmental stages enhanced plant tolerance to Zn^2+^ [65]. Additionally, Zn^2+^ treatment strongly activated the promoter activity of *RPS15A*, which played a crucial role in maintaining reactive oxygen species (ROS) balance during oxidative stress [66, 67]. The heavy metal transporter *OsNRAMP1* modulated disease resistance in rice by regulating ROS homeostasis [68]. Zn^2+^ could effectively delay tobacco mosaic virus (TMV) replication and movement in *Nicotiana benthamiana* [69]. Collectively, these results suggested a potential role of Zn^2+^ ions in banana root tips responding to *Foc* TR4 infection.

Notably, the *MaADH3*/*MaACA7* module, identified in the pericycle that containing the highest number of DEGs responding to *Foc* TR4 infection, was zinc ion-dependent (Fig. 7D-E). Zn^2+^ acted as a cofactor by coordinating with the imidazole ring of histidine in ACA enzymes and the thiol group of cysteine in ADH enzymes to form the catalytic center, which was essential for maintaining the structural integrity and catalytic activity of these enzymes [70, 71]. In studies on the acute zinc deficiency response in sorghum, the expression and activity of *SbCA* recovered immediately after zinc supplementation, whereas *SbADH* showed a delayed recovery, suggesting that *SbCA* responded more rapidly to zinc signaling than *SbADH* [72]. In rice, Zn^2+^ deficiency reduced ADH activity and ATP production, leading to decreased root metabolic activity [73]. *AtTIP2–2* has been shown to involve in Zn^2+^ sequestration in roots [74]. In this work, *TIP2–2*, the homolog of *AtTIP2–2*, showed upregulated expression in pericycle cells at the terminal stage of the differentiation trajectory (Fig. 5E). Therefore, we speculated that *TIP2–2* might participate in Zn^2+^ transport, facilitating the activation of Zn^2+^ binding sites in MaACA7 and MaADH3. In summary, we highlighted a critical role of Zn^2+^ ions in pericycle cells responding to Fusarium wilt.

Metal ions, as trace elements, have disease-responsive mechanisms that are difficult to be detected by using bulk RNA-seq, whereas single-cell transcriptomics is more effective in uncovering these fine molecular mechanisms [75]. Currently, single-cell sequencing technology has been widely applied in various research areas, including plant development, stress responses, and pathogen interactions. In rubber tree, *HbCNL2* positively regulated defense against powdery mildew in epidermal cells of leaves [76]. The coleoptile of wheat roots jointly responded to *Fusarium graminearum* infection, highlighting the cooperative mechanisms between different cell types during fungal invasion [77]. In maize leaves, guard cells and epidermal cells exhibited an immune state even before infection, highlighting the role of ZmChit7 protein in resistance to Puccinia polysora [78]. Likely, maize root apical meristem (*ZmWOX5b* and *ZmPIN1a*) and phenylpropanoid (*ZmPAL6*, *ZmCCoAOMT2*, and *ZmCOMT*) related genes in the cortex, columella, and vascular cells collaboratively constructed a cell-specific immune regulatory network in response to *Fusarium verticillioides* [18]. Together, these results suggest that single-cell transcriptomics provide important insights into the heterogeneous responses of different cell types in plant disease resistance.

In this work, we reported a single-cell transcriptomic atlas of banana root tips responding to Fusarium wilt. We analyzed cell-specific marker genes, cell differentiation trajectories, *Foc* TR4-responsive genes, and cell-specific regulatory networks. We also revealed a crucial role of *MaKAN4* in banana disease resistance by regulating the Zn^2+^-dependent *MaACA7*/*MaADH3* module. These findings provide new insights into the molecular mechanisms underlying the response of banana root tips to *Foc* TR4 infection.

## Materials and methods

### Plant materials and fungal inoculation

The banana cultivar ‘Cavendish’ (*Musa acuminata* L., AAA group) was provided by the Institute of Tropical Bioscience and Biotechnology, Chinese Academy of Tropical Agricultural Sciences. Banana seedlings with five fully-developed leaves were prepared for subsequent experiments. After inoculation, the plants were incubated in a constant-temperature growth chamber at 28°C with a 16 h light/8 h dark photoperiod and a light intensity of 5000 lx.

The GFP-labeled *Fusarium oxysporum* f. sp. *cubense* tropical race 4 (*Foc* TR4) strain was provided by Dr. Yufeng Chen from the Chinese Academy of Tropical Agricultural Sciences. Freshly cultured *Foc* TR4-GFP mycelia were inoculated onto PDA solid medium and incubated at 28°C for 5 days. Spores were washed with sterile water, counted using a hemocytometer, and 100 mL of spore suspension was introduced into the soil to achieve a final concentration of 1.0 × 10^5^ cfu/g soil.

### Laser confocal and microscopic observations

For microscopic examination, banana roots were washed with sterile distilled water and observed under a confocal laser scanning microscope (OLYMPUS, FV3000) equipped with filter blocks matching the spectral characteristics of green fluorescent protein (488 nm) and root autofluorescence (543 nm and 595 nm) [79].

Root tips (5 mm) of banana plants after *Foc* TR4 inoculation at 21 days were excised in formaldehyde-acetic acid, dehydrated through an ethanol gradient and Van-Clear (Huntz, Wuhan, China), embedded in paraffin, and mounted on microscope slides. Sections from the control and *Foc* TR4-treated groups were stained with 0.01% toluidine blue, quickly dehydrated, sealed with a resinous mounting medium, and observed under a light microscope (Leica DM6 B Upright LED Fluorescence Microscope).

### Bulk RNA-seq and analysis

Root tips (5 mm) of banana plants after *Foc* TR4 inoculation at 0, 7, 14, 21, and 28 days were used for bulk transcriptome sequencing. RNA-seq was performed as previously described [80]. Total RNA was extracted using the RNeasy Plus Mini Kit (Qiagen), and its quality was assessed with Qubit RNA Assay Kit, gel electrophoresis, and Agilent Bioanalyzer 2100. mRNA was purified with oligo(dT)-attached beads. Libraries were prepared using the Nextera XT Kit (Illumina) and sequenced on the NovaSeq6000 platform. Reads were trimmed with fastp v0.23.0 [81] and aligned to the Cavendish genome using HISAT2 [82]. Gene expression was calculated using HTSeq [83].

### ScRNA-seq library preparation and sequencing

Cavendish root tip samples were carefully crushed into small pieces. The nuclei isolation buffer (NIB) was freshly prepared with the following composition: 5% Dextran T40, 0.4 M sucrose, 10 mM MgCl₂, 1 mM dithiothreitol (DTT), 2 U/μL RNase inhibitor, 0.1% Triton X-100, and 100 mM Tris–HCl (pH 7.4). The root tip fragments were transferred into centrifuge tubes containing NIB and centrifuged at 300 × g/min. The supernatant was collected and filtered through a 40 μm cell strainer into a new 15 mL centrifuge tube. The filtrate was centrifuged at 2000 × g for 5 minutes at 4°C, and the supernatant was discarded. The nuclei pellet was resuspended in Wash Buffer. Single cell suspensions of banana root tips were mixed with 0.4% Trypan Blue dye and counted using the Countess® II Cell Counter. The viable cell concentration was adjusted to 1000–2000 cells/μL. Gel beads with barcode information were combined with the cell mixture and separated into GEMs (Gel Beads-In-Emulsions) by oil. The gel beads released barcode sequences, which were reverse transcribed into cDNA and labeled. After PCR amplification, the products were pooled to construct the sequencing library. The library was sequenced using the PE150 mode on the Illumina platform [84].

### Differentially expressed genes (DEGs)

Differential expression analysis was carried out using DESeq2 [85]. The significance of differential gene expression was assessed based on the Wald test within a negative binomial distribution model. DEGs were identified by setting the false discovery rate (FDR) ≤0.05 and the absolute fold change ≥2. GO and KEGG clustering analysis were performed using the online platform (https://www.omicsmart.com/).

### Cell clustering and identification of marker genes

Seurat and UMAP were used to reduce the dimensionality of 48 983 cells [86, 87]. An SNN graph was constructed based on Euclidean distance in PCA space, with edge weights adjusted using Jaccard distance to refine local neighborhood overlap. Cells were then clustered using the Louvain method to optimize modularity.

Marker genes were selected based on the following criteria: expressed in over 40% of target cells within the cluster, with an average log2 fold change >1 and P < 0.01. The selected markers were aligned with known Arabidopsis marker genes (E < 10^−5^) and further validated by RNA *in situ* hybridization.

### RNA *in situ* hybridization

Banana root tips (5 mm) infected with *Foc* TR4 were collected at 21 days post-inoculation and fixed in FAA solution (10% formaldehyde, 5% acetic acid, 47.5% ethanol) for 24 hours. After dehydration, permeabilization, wax infiltration, embedding, sectioning, and mounting, probes of four marker genes were synthesized (Supplemental Table S12), and RNA *in situ* hybridization were performed [88].

### Pseudotime analysis

Pseudotime analysis was conducted using Monocle2 [89]. Variable genes were selected to define the developmental trajectory and DDRTree was used for dimensionality reduction. The trajectory and dynamic genes were visualized with plot_cell_trajectory and plot_pseudotime_heatmap. Gene modules were identified by hierarchical clustering based on Euclidean distance and complete linkage, which clustered genes with similar pseudotime expression dynamics. Branch-dependent genes were identified and visualized using differentialGeneTest and plot_genes_branched_heatmap, respectively.

### Weighted gene co-expression network analysis (WGCNA) within cell types

Co-expression analysis was performed using the WGCNA package in R to detect the relative relationship between genes and cell types [90]. A weighted adjacency matrix was created using unsupervised hierarchical clustering, with the soft threshold power setting to six to analyze scale-free topology. Modules were identified setting minModuleSize = 50 and similarity = 0.85. Key transcription factors and genes were screened based on gene expression differences and connectivity, using Z-score normalization.

### Yeast one hybrid assay

The transcription factor was cloned into the pGADT7 vector, and promoter fragments were inserted into the pAbAi vector. Promoter constructs were integrated into Y1HGold yeast, followed by transformation with MaKAN4-pGADT7, and interactions were assessed on SD/-HLT/3AT medium.

### Dual luciferase assay

Promoter fragments were cloned into the pGreenII 0800-LUC reporter vector, and *MaKAN4* coding sequences were inserted into the pGreenII 62-SK effector vector. Both constructs were co-infiltrated into Nicotiana benthamiana leaves. After 48 hours, LUC and REN activities were measured, and the LUC/REN ratio was used to assess promoter activities. Statistical significance was determined using Student’s t-test.

### Zn^2+^ treatment and gene expression analysis by RT-qPCR

Banana seedlings inoculated with *Foc* TR4 were treated with various ZnSO₄·7H₂O solutions with final concentrations of 15 μM, 45 μM, and 75 μM Zn^2+^. Zn^2+^ solutions were applied by root drenching once per week. Control plants received the same volume of distilled water. Phenotypic observations were recorded at 21 DPI under identical growth conditions. At the same time, total RNA was extracted from banana root tips, reverse-transcribed into cDNA, and analyzed by RT-qPCR using SYBR Green. Actin was used as the internal control, and relative gene expression was calculated using the 2^-ΔΔCt^ method. Statistical differences among groups were evaluated using ANOVA, followed by Tukey’s honestly significant difference test for multiple comparisons. Groups not sharing the same lowercase letter were considered significantly different at P < 0.05.

## Supplementary Material

Web_Material_uhaf220

## Data Availability

The dataset has been uploaded to National Genomic Data Center (BioProject: PRJCA038648).
