# Supplementary figures and images for: Single-cell transcriptome atlas unveils transcriptional regulation networks of banana root tips in response to Fusarium oxysporum infection
Source: Hortic Res. 2025 Aug 22;12(11):uhaf220. doi: 10.1093/hr/uhaf220 (PMC12598465; doi:10.1093/hr/uhaf220)

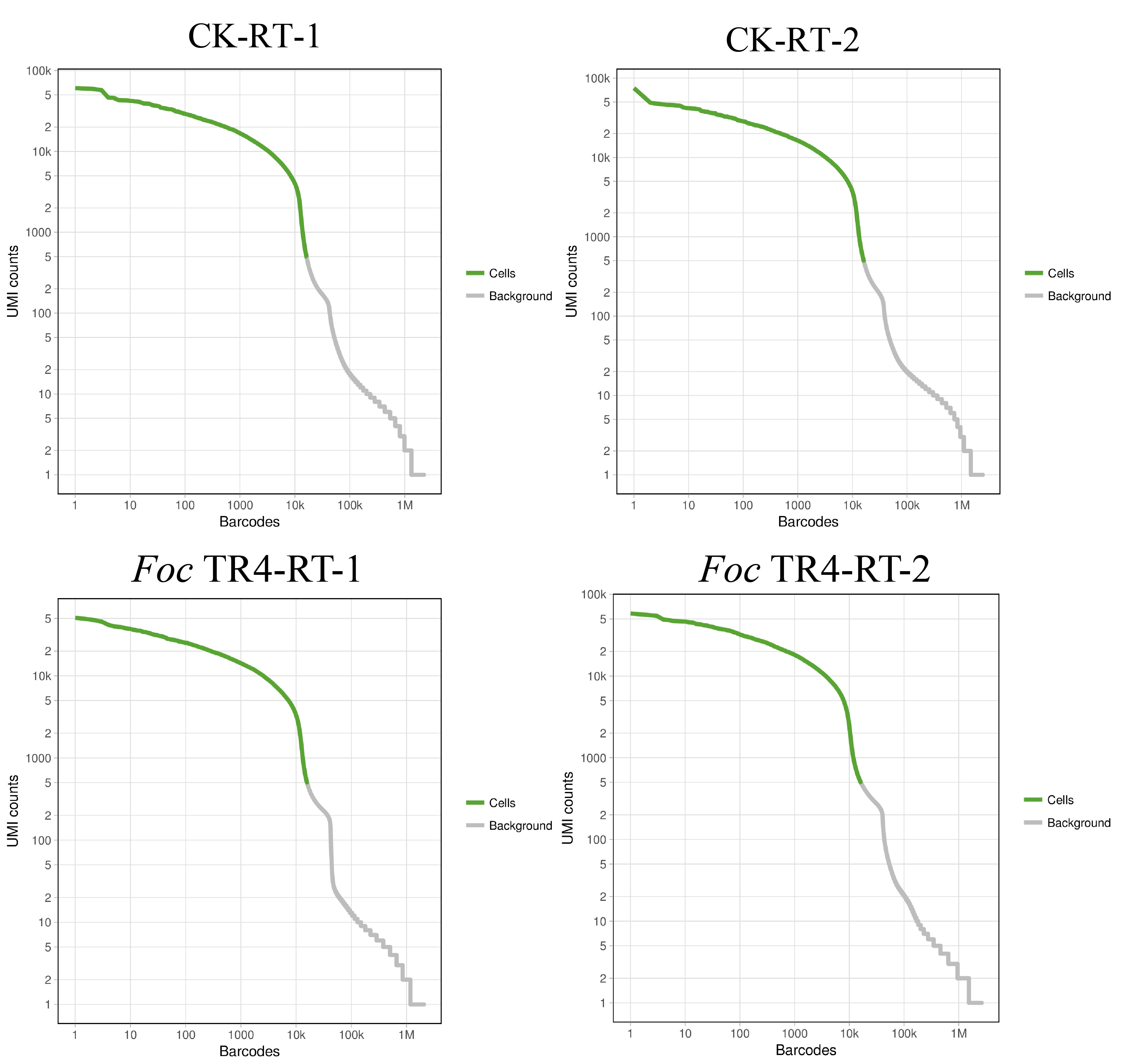

Supplement: Web_Material_uhaf220 [file web_material_uhaf220.zip › Supplementary Fig S1.jpg]

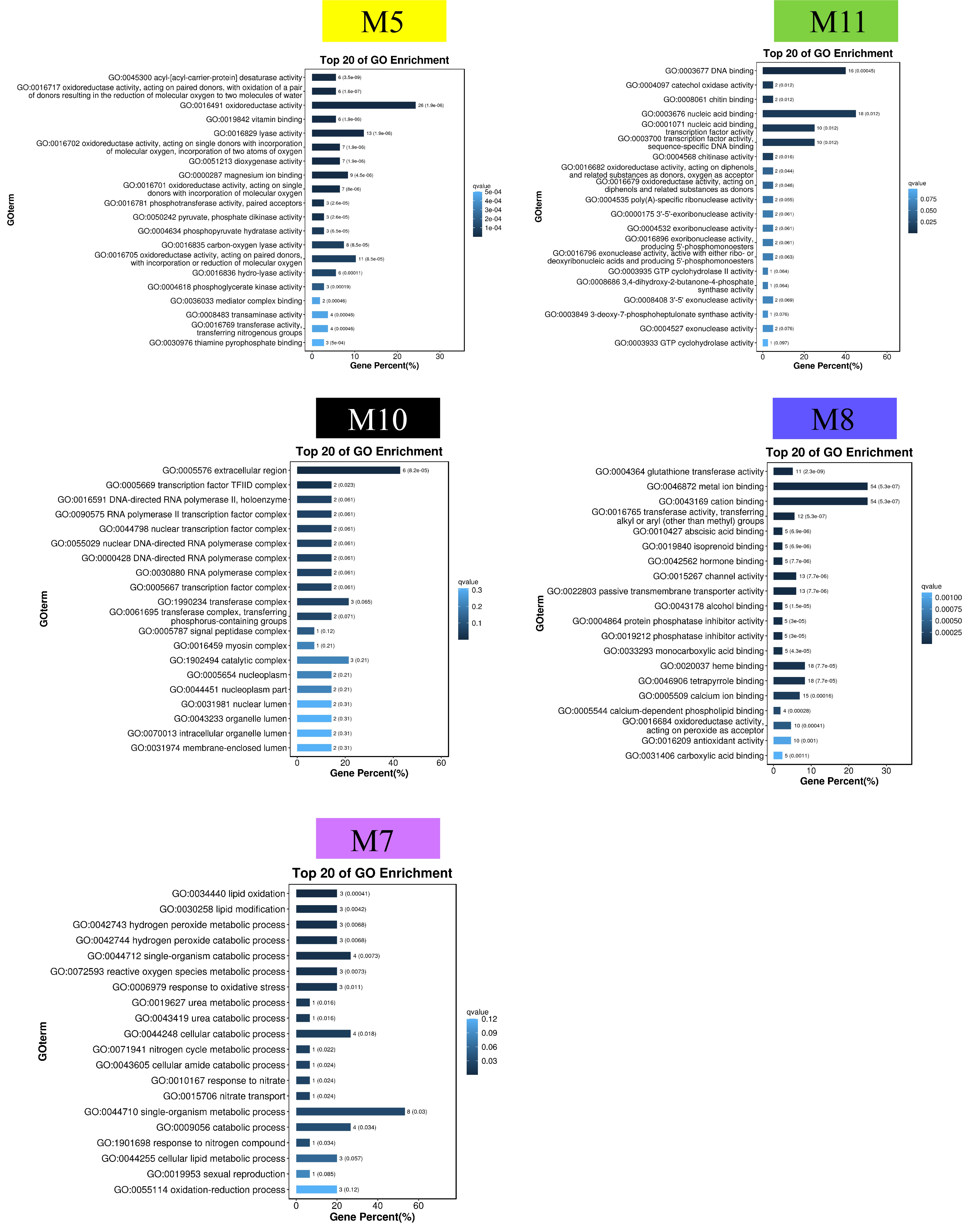

Supplement: Web_Material_uhaf220 [file web_material_uhaf220.zip › Supplementary Fig S10.jpg]

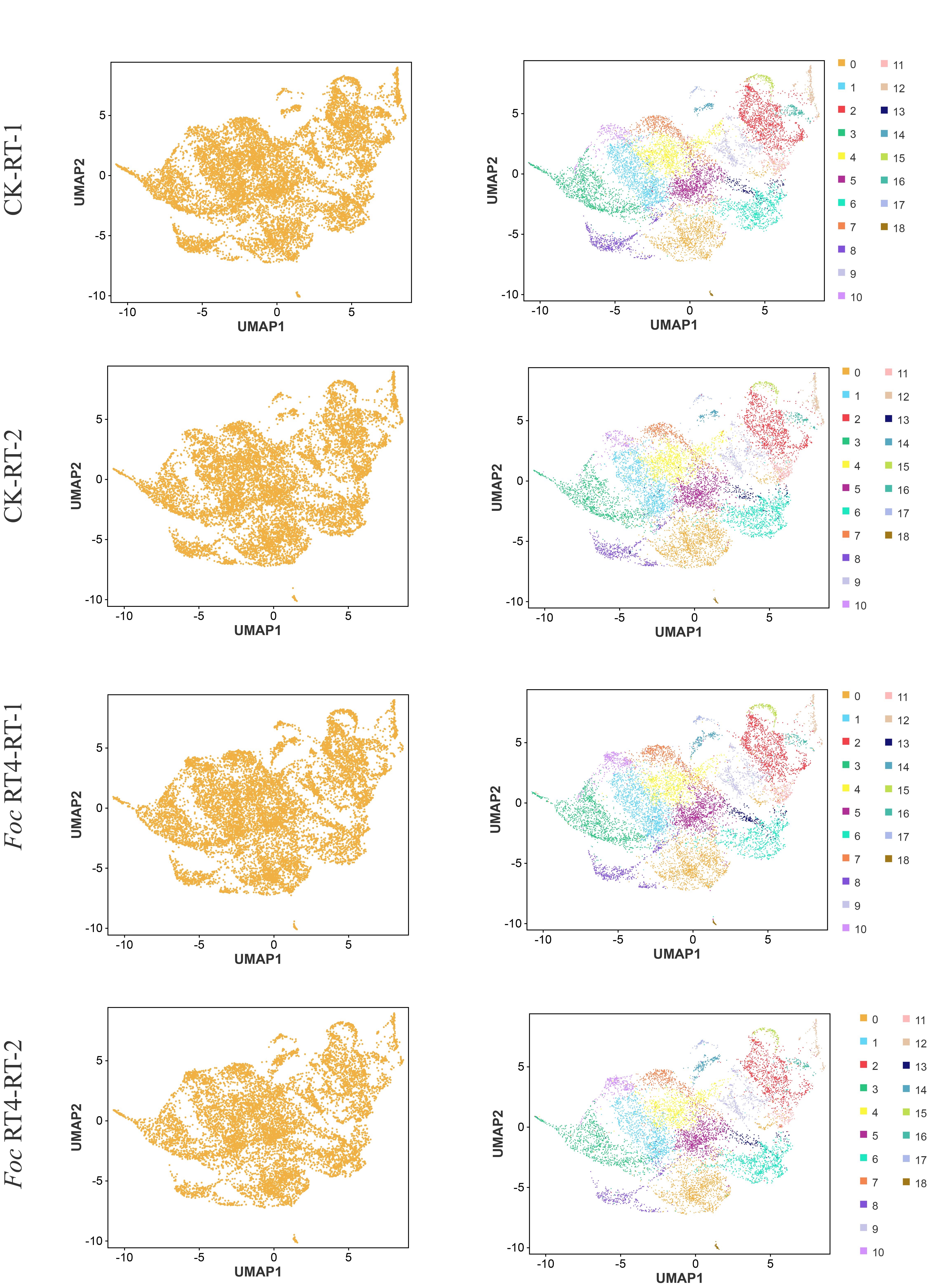

Supplement: Web_Material_uhaf220 [file web_material_uhaf220.zip › Supplementary Fig S2.jpg]

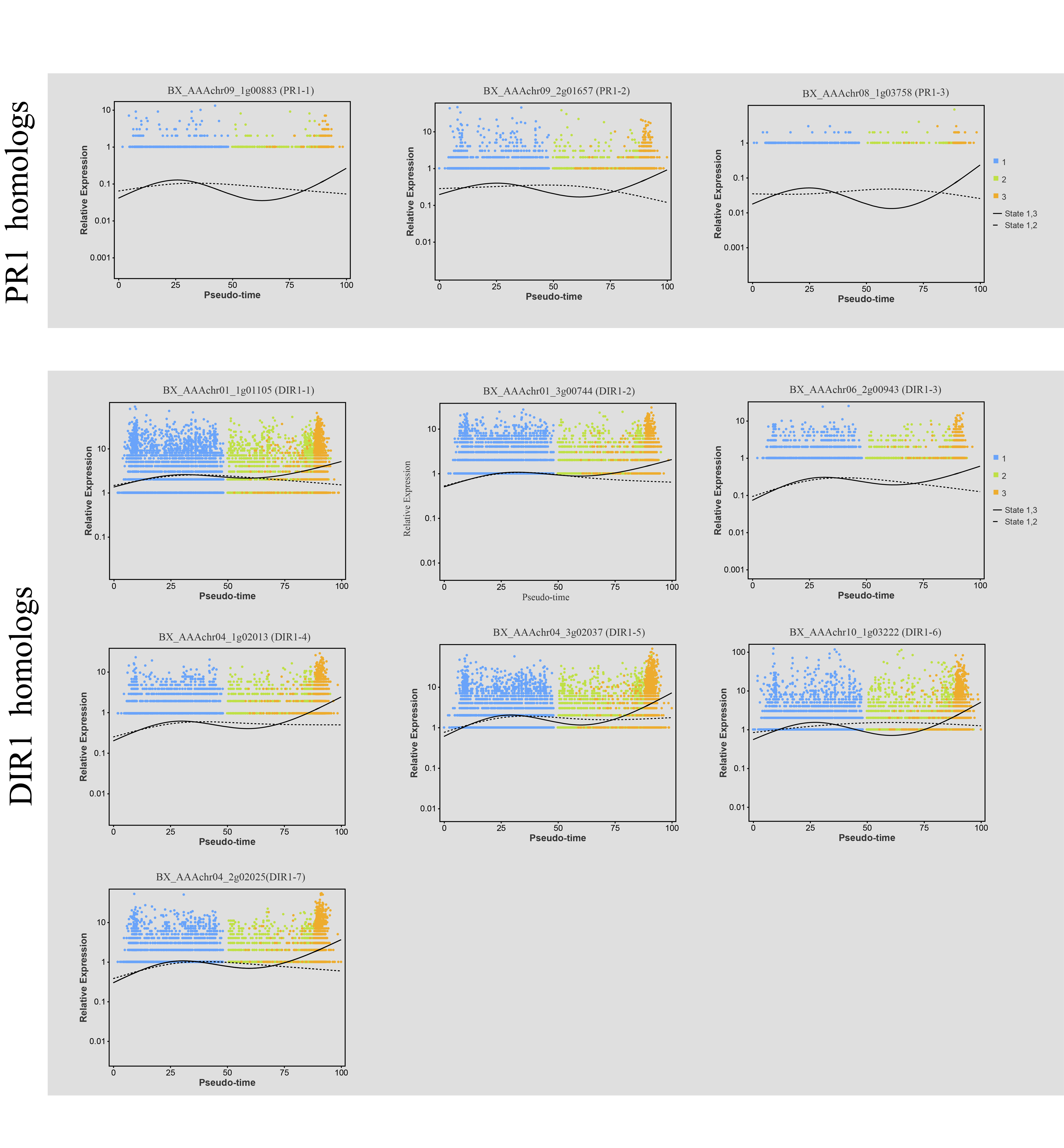

Supplement: Web_Material_uhaf220 [file web_material_uhaf220.zip › Supplementary Fig S3.jpg]

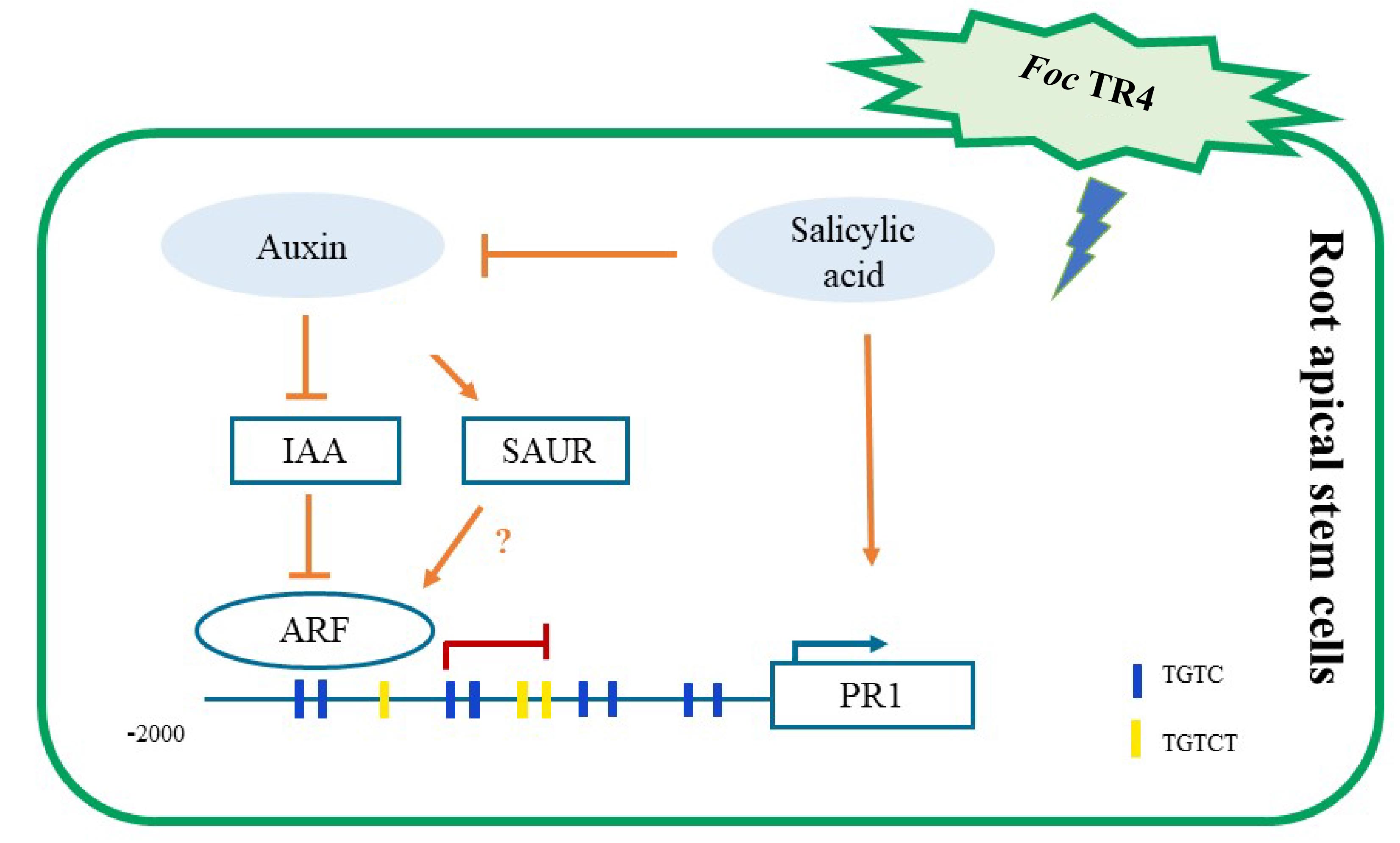

Supplement: Web_Material_uhaf220 [file web_material_uhaf220.zip › Supplementary Fig S4.jpg]

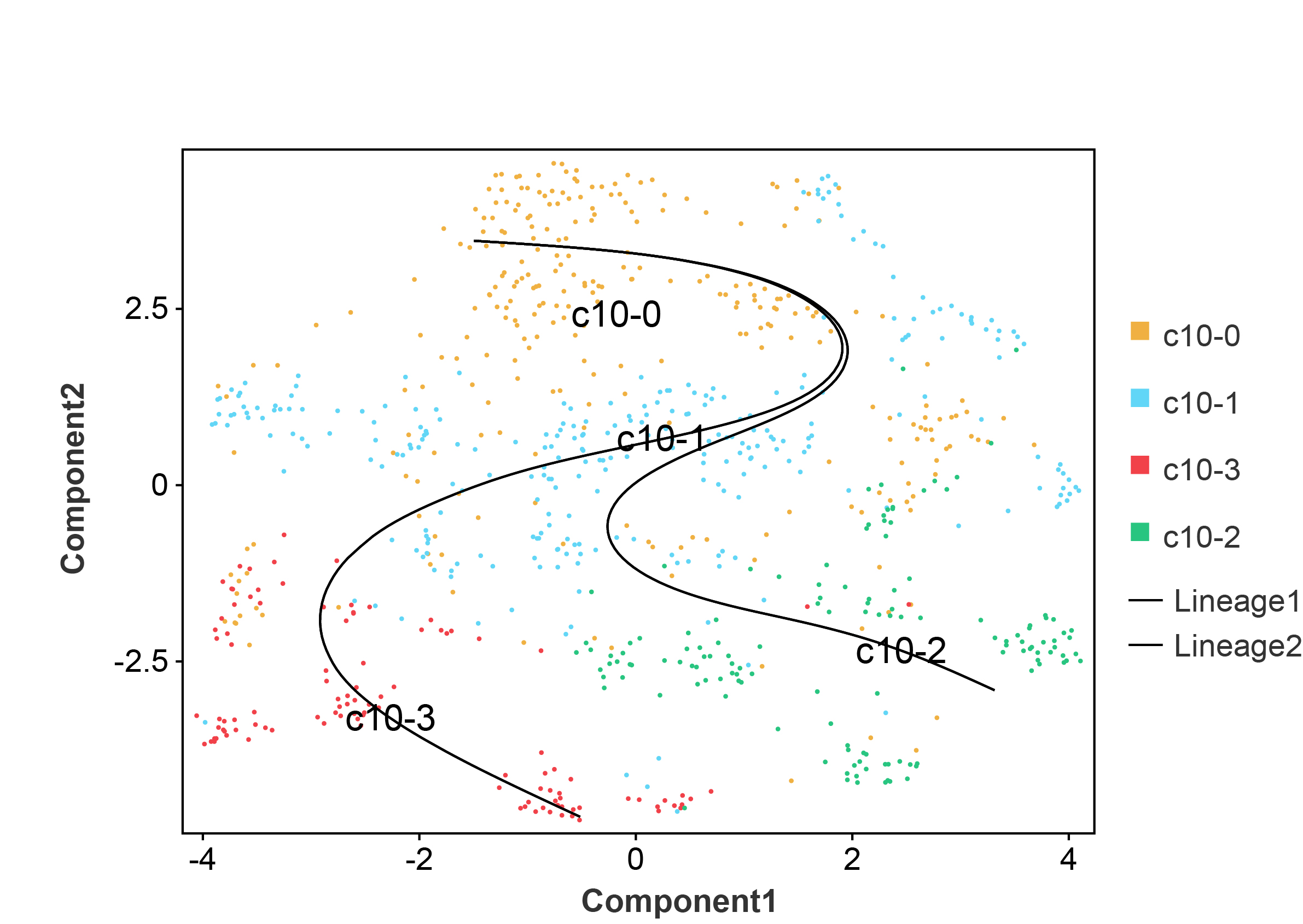

Supplement: Web_Material_uhaf220 [file web_material_uhaf220.zip › Supplementary Fig S5.jpg]

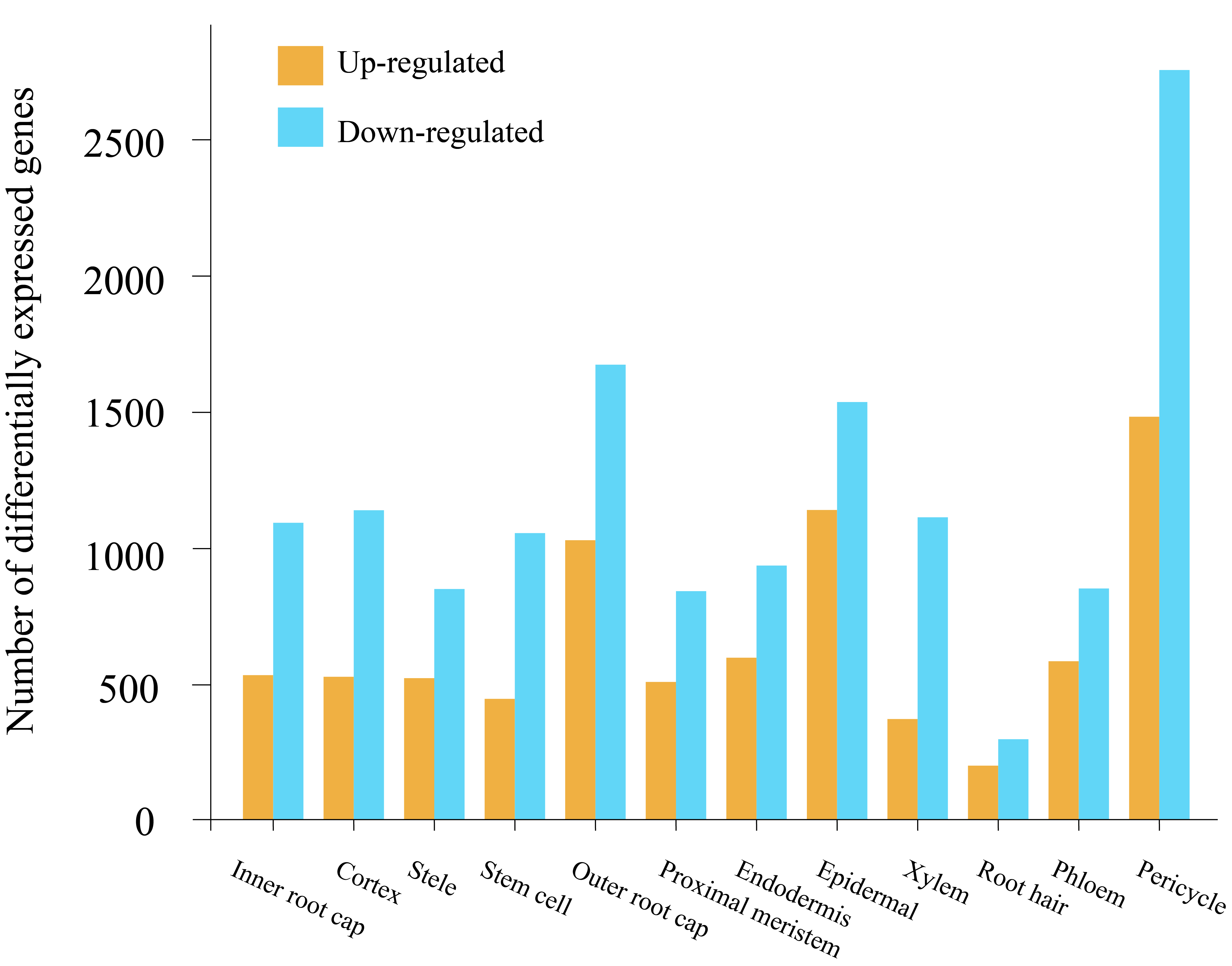

Supplement: Web_Material_uhaf220 [file web_material_uhaf220.zip › Supplementary Fig S6.jpg]

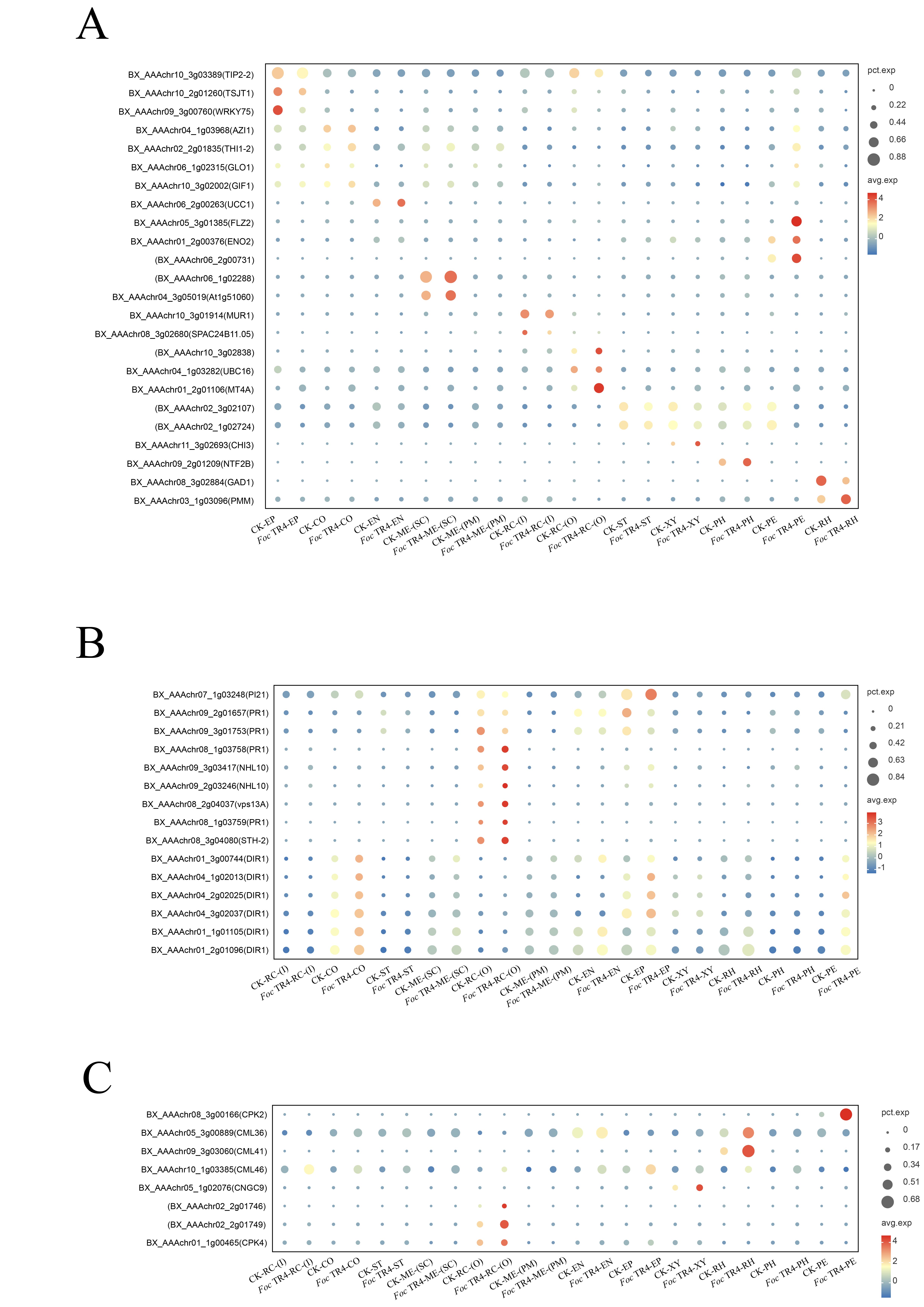

Supplement: Web_Material_uhaf220 [file web_material_uhaf220.zip › Supplementary Fig S7.jpg]

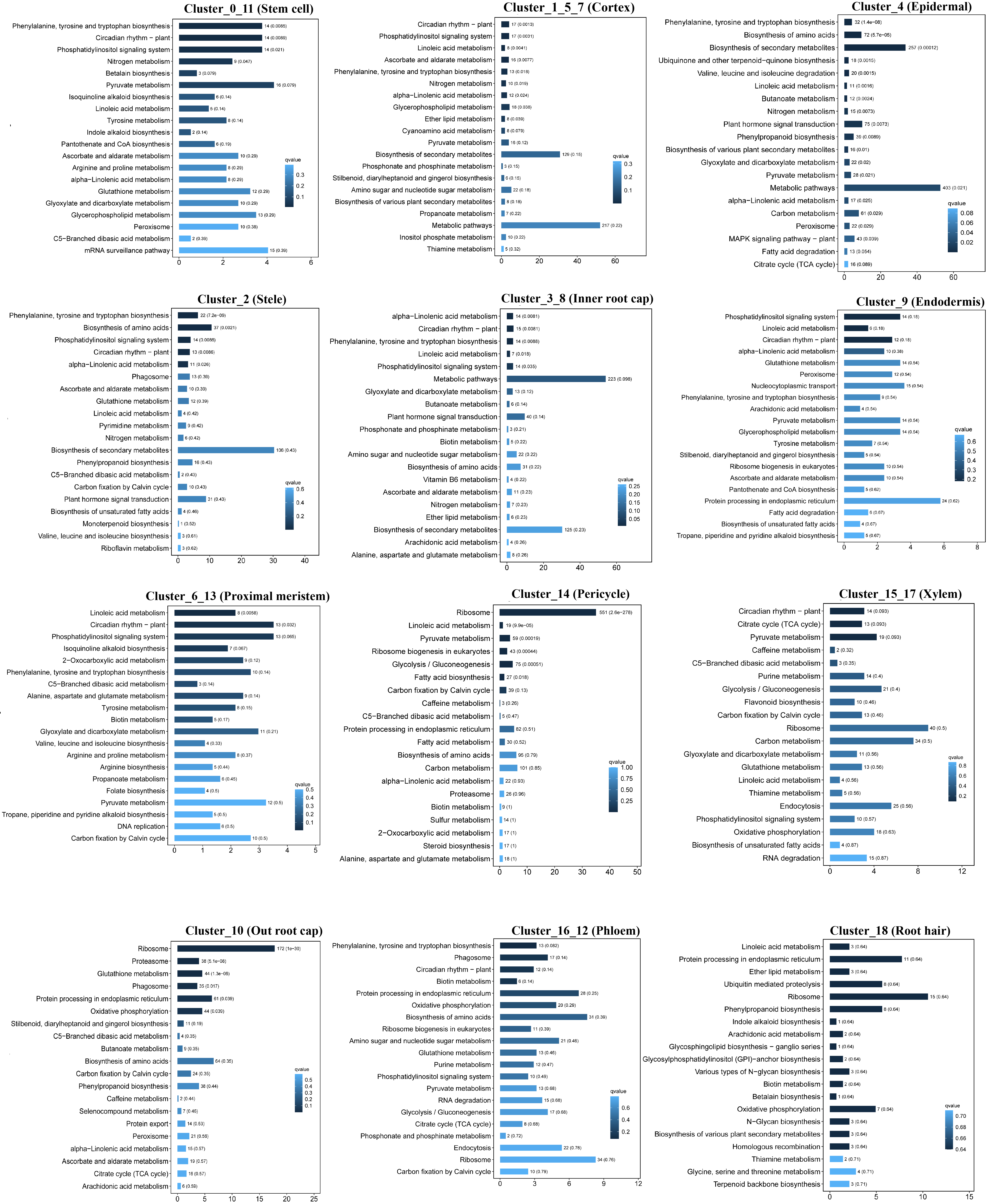

Supplement: Web_Material_uhaf220 [file web_material_uhaf220.zip › Supplementary Fig S8.jpg]

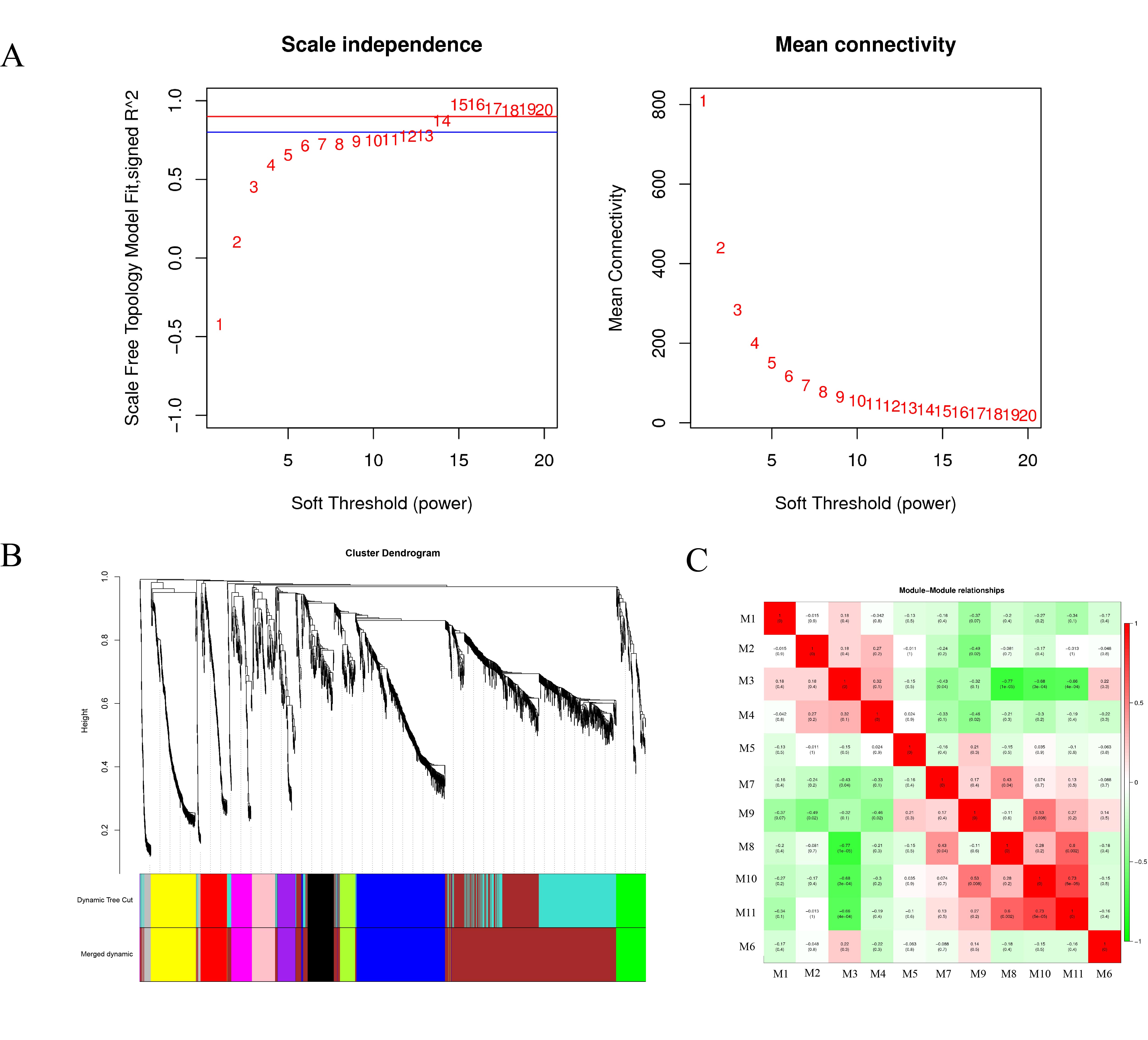

Supplement: Web_Material_uhaf220 [file web_material_uhaf220.zip › Supplementary Fig S9.jpg]
